# A Potential Negative Regulatory Function of Myostatin in the Growth of the Pacific Abalone, *Haliotis discus hannai*

**DOI:** 10.3390/biology12010014

**Published:** 2022-12-21

**Authors:** Jianfang Huang, Mingcan Zhou, Jianming Chen, Caihuan Ke

**Affiliations:** 1 Institute of Oceanography, College of Geography and Oceanography, Minjiang University, Fuzhou 350108, China; 2 College of Ocean and Earth Sciences, Xiamen University, Xiamen 361102, China; 3 Fujian Key Laboratory of Genetics and Breeding of Marine Organisms, Xiamen University, Xiamen 361102, China

**Keywords:** myostatin, *Haliotis discus hannai*, molluscan growth, RNA interference, single nucleotide polymorphism

## Abstract

**Simple Summary:**

The *Haliotis discus hannai* (*H. discus hannai*) is one of the most economically important species cultured in China. Currently, the problems of slow growth and small abalone sizes have become increasingly serious, causing economic losses to farmers. The most effective way to solve the problems of slow growth, miniaturization, and lack of large abalone is by molecular genetic manipulation to breed high-quality abalone that have rapid growth rates. Analyzing the genetic mechanism of abalone growth and discovering key functional genes are thus critical for this genetic improvement program. Many studies have confirmed that myostatin is an important regulator of muscle growth in animals. In this study, we investigated the molecular structure and function of *hdh-myostatin* in *H. discus hannai*. The results revealed that hdh-myostatin contained structural characteristics typical of the TGF-β superfamily and was involved in the regulation of growth. Our findings would help to clarify the role of *hdh-myostatin* in the regulation of abalone growth and provide a reference for the application of molecular markers of growth traits in mollusk breeding.

**Abstract:**

Myostatin, also known as GDF8, is a member of the transforming growth factor-β (TGF-β) superfamily. In vertebrates, myostatin negatively regulates the growth of skeletal muscle. In invertebrates, it has been reported to be closely related to animal growth. However, knowledge concerning the molecular mechanisms involved in the myostatin regulation of molluscan growth is limited. In this study, we found that the hdh-myostatin open reading frame (ORF) comprised 1470 base pairs that encoded 489 amino acids and contained structural characteristics typical of the TGF-β superfamily, including a C-terminal signal peptide, a propeptide domain, and TGF-β region. Gene expression analysis revealed that *hdh-myostatin* mRNA was widely expressed at different levels in all of the examined tissues of *Haliotis discus hannai*. Nine single nucleotide polymorphisms (SNPs) were associated with the growth traits. RNA interference (RNAi) against *hdh-myostatin* mRNA significantly downregulated *hdh-myostatin* at days 1, 15, and 30 post injection, and the pattern was correlated with downregulation of the genes TGF-β receptor type-I (*hdh-T*β*R* I), activin receptor type-IIB (*hdh-ActR* IIB), and mothers against decapentaplegic 3 (*hdh-Smad*3). After one month of the RNAi experiment, the shell lengths and total weights increased in the abalone, *Haliotis discus hannai*. The results of qRT-PCR showed that the *hdh-myostatin* mRNA level was higher in the slow-growing group than in the fast-growing group. These results suggest that hdh-myostatin is involved in the regulation of growth, and that these SNPs would be informative for further studies on selective breeding in abalone.

## 1. Introduction

Abalone is an important mariculture mollusk in China. The species *Haliotis discus hannai* (*H. discus hannai*) is one of the most economically important species cultured in China [1,2], and Fujian Province accounts for nearly 80% of total abalone production in China [3,4]. However, with the rapid development of the abalone breeding industry, the gap between the abalone germplasm quality and production requirements has become increasingly prominent. On the one hand, the problems of slow growth and small abalone size have become increasingly serious, causing economic losses to farmers. On the other hand, the domestic large abalone market has long been monopolized by foreign wild abalone. These problems have created a “bottleneck” restricting the healthy development of the abalone aquaculture industry in China. At present, the most effective way to solve the problems of slow growth, miniaturization, and lack of large abalone is through molecular genetic manipulation to breed high-quality abalone that have rapid growth rates. Analyzing the genetic mechanism of abalone growth and discovering key functional genes are thus critical for this genetic improvement program.

Hybridization is an effective method for genetic improvement in aquaculture that can introduce improved traits to the hybrids [5,6]. *H. fulgens* was introduced from the United States [7], with a fast growth rate. You et al. (2015) have cultivated the Lvpan abalone (*H. discus hannai ♀ × H. fulgens ♂*) with fast growth and large size through interspecific crossbreeding technology [8]. It has gradually become a new breed of abalone in Fujian Province [8]. Although the appearance of the Lvpan abalone alleviates the demand of the industry, the mechanism for the rapid growth of the abalone is still unclear. Lvpan abalone may be an ideal material for studying growth traits of abalone and can be used to prove the action mechanism of key genes related to abalone growth. It is helpful to further guide the cultivation of new species with rapid growth.

Myostatin, also known as growth differentiation factor 8 (GDF8), belongs to the transforming growth factor β (TGF-β) superfamily [9]. Many studies have confirmed that myostatin has an important role in animals’ muscle growth. As a secreted glycoprotein, it has similar amino acid structural characteristics to TGF-β superfamily members, including an N-terminal signal peptide, a glycosylation site, a protease hydrolysis site, and nine conserved C-terminal cysteine residues [10,11]. In mammals, the deletion of myostatin leads to a dramatic increase in skeletal muscle mass [12]. In fish, the absence or blockage of myostatin in early developmental stages has also produced giant phenotypes [13,14]. In addition to what has been observed in vertebrates, a significant increase in muscle cellularity has been induced by RNA interference (RNAi) of myostatin in *Patinopecten yessoensis* [14]. In mollusks, myostatin has been characterized in species such as *Argopecten irradians* [15], *Chlamys farreri* [16], *Mytilus chilensis* [17], *Argopecten purpuratus* [18], *Sinonovacula constricta* [19], *Chlamys nobilis* [20], *H. rufescens* [21], and *H. diversicolor supertexta* [10]. Myostatin has been demonstrated to function via forming dimers with two types of membrane-bound receptors, type I receptors (including ALK4, ALK5, TβR I, and ActR IB), and type II receptors (including ActR II, ACVR II, ActR IIB, and ACVR IIB) [11,22]. The myostatin initially binds to type II receptors, and the type II receptors subsequently phosphorylate type I receptors, leading to downstream signaling, including the phosphorylation of drosophila mothers against decapentaplegic proteins (Smads) (including Smad2 and Smad3) [23]. However, studies on the function of myostatin involved in the growth of *H. discus hannai* are still scarce.

In vertebrates, myostatin negatively regulates skeletal muscle growth [24]. In invertebrates, it has also been reported to be closely related to animal growth [25,26]. In this paper, we cloned the ORF sequence of *hdh-myostatin* in *H. discus hannai* and analyzed its sequence structure. Secondly, the expression characteristics of *hdh-myostatin* at different developmental time points and tissues in *H. discus hannai* were analyzed by qRT-PCR. Thirdly, we examined the expression changes of these genes (*hdh-myostatin*, *hdh-TβR* I, *hdh-ActR* IIB, *hdh-Smad*3, and *MHC*) by RNAi to clarify the role of *hdh-myostatin* in abalone growth. Finally, we verified the relationship between myostatin and growth in Lvpan abalone and detected SNPs associated with abalone growth. Our research will provide theoretical support for the cultivation of new high-quality abalone species with the ability of rapid growth and large size and have important practical significance for promoting the transformation and upgrading of abalone aquaculture.

## 2. Materials and Methods

### 2.1. Experimental Animals and Sample Collection

All *H. discus hannai* and hybrid Lvpan abalones were obtained from Fuda Abalone Aquaculture Co., Ltd. in Jinjiang, Fujian province, China. The tissues of adductor muscle of *H. discus hannai* from different developmental time points (one month (1M), four months (4M), seven months (7M), 10 months (10M), 12 months (12M), 14 months (14M), 16 months (16M), and 18 months (18M)) were collected, each with three replicates. Six *H. discus hannai* were sacrificed, and the lymphocytes, gonad, gill, mantle, cerebral ganglion, hepatopancreas, adductor muscle, and foot tissues were collected. Twelve of the larger *H. discus hannai* (“L-DD-group”; mean total weight, 8.88 ± 0.79 g; about one year old), 12 of the smaller *H. discus hannai* (“S-DD-group”; mean total weight, 2.30 ± 0.42 g; about one year old), 12 of the larger Lvpan abalone (“L-DF-group”; mean total weight, 9.02 ± 0.74 g; about one year old), and 12 of the smaller Lvpan abalones (“S-DF-group”; mean total weight, 2.49 ± 0.54 g; about one year old) were sacrificed for the adductor muscles. All of the samples were collected and frozen immediately in liquid nitrogen before being stored at −80 °C for subsequent experiments.

### 2.2. RNA Isolation and cDNA Synthesis

The total RNA isolation and the cDNA template synthesis followed the methods previously described by Sun et al., (2020) [14].

### 2.3. Hdh-Myostatin ORF Confirmation and Sequence Analysis

Using the genome of *H. discus hannai*, *hdh-myostatin-*F and *hdh-myostatin-*R (Table 1) were designed for the amplification of the *hdh-myostatin* open reading frame (ORF). The corrected ORF sequences were uploaded to GenBank (accession number: OP856630). The deduced amino acid sequence of the hdh-myostatin protein was obtained by Lasergene software. The signal peptide of the hdh-myostatin protein was predicted using the SingalP 5.0 Server. The proteolytic processing site was predicted using the Prop 1.0 server. The secondary structure of the hdh-myostatin protein was analyzed by SOPMA. The protein domains of the hdh-myostatin protein were analyzed using CDD. We downloaded myostatin protein sequences of different species from the NCBI database and compared them using the ClustalW2 program. After that, we constructed a myostatin phylogenetic tree with the neighbor-joining algorithm using the MEGA program.

### 2.4. Sequence and SNP Analysis

A total of 222 *H. discus hannai* selected from 10 families were used for the verification of single nucleotide polymorphisms (SNPs) [27]. The foot muscle of abalones was obtained for DNA extraction using a DNeasy 96 Blood and Tissue Kit (Qiagen, Shanghai, China). Five growth-related traits (shell length, shell width, total weight, and muscle weight) were measured to represent the phenotype. The ORF sequences of hdh-myostatin from sampled *H. discus hannai* were genotyped by resequencing with a filter for SNPs with a minor allele frequency less than 10%, and the genome of *H. discus hannai* was utilized as a reference. Polymorphic information content (PIC) of SNPs was evaluated using POPGENE 1.32 software according to the program instructions.

### 2.5. RNA Interference of Hdh-Myostatin

A 388 bp fragment of hdh-myostatin and 497 bp of the green fluorescent protein (EGFP) gene (an exogenous control gene) were amplified using specific primers (Table 1). The dsRNA targeting hdh-myostatin was acquired following the methods previously described by Sun et al., (2020) with slight modifications [14].

About 80 *H. discus hannai* (~4–5 g) were selected and randomly divided into two groups (the *EGFP* control group and the *hdh-myostatin* RNAi experimental group). The injection solution was prepared by diluting the purified dsRNA in filter-sterilized seawater. The abalones were injected with the purified dsRNA at a dose of 100 μg per abalone once a week for a total of five times. Finally, samples of the cerebral ganglion and adductor muscle were collected at days 1, 15, and 30, and RNA was isolated for qRT-PCR. The growth traits of *H. discus hannai* in each group were measured at the end of the experiment.

### 2.6. Real-Time Quantitative Reverse Transcription PCR

The gene expression levels of hdh-myostatin, *hdh-TβR* I (TGF-β receptor type-I), *hdh-ActR II*B (activin receptor type-IIB), *hdh-Smad*3 (mothers against decapentaplegic 3), and *MHC* (myosin heavy chain) were determined by qRT-PCR. The gene-specific primers are listed in Table 1. Relative gene expression levels were quantified based on *β-actin* and *18S rRNA* using the 2^−∆∆CT^ method. The PCR amplification was performed following the methods previously described [28].

### 2.7. Statistical Analysis

All data were presented as mean ± standard deviation (SD). The statistical analysis employed one-way ANOVA with Duncan’s tests or *t*-tests using SPSS 19.0. The significance level for the analysis was specified as *p* < 0.05.

## 3. Results

### 3.1. Characterization of Hdh-Myostatin

**Figure 1:**
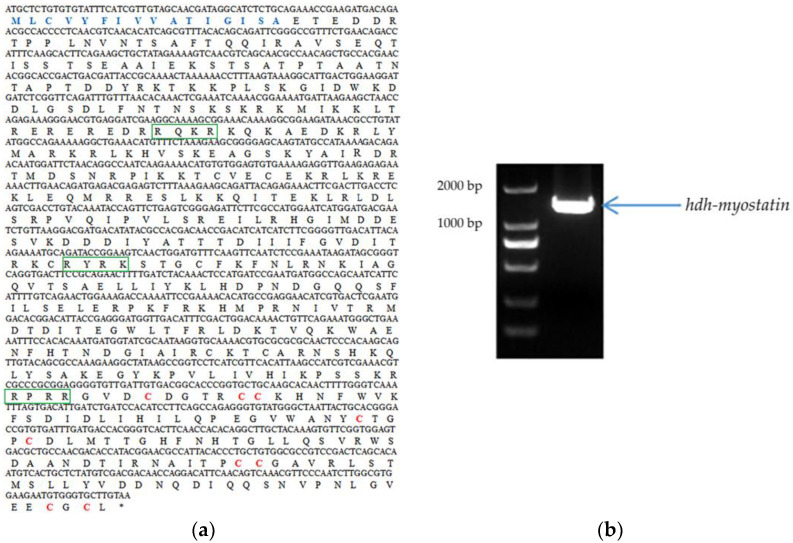
Gene information for hdh-myostatin from *Haliotis discus hannai*. (**a**) The putative signal peptide is shown in blue. The three proteolytic processing sites are denoted by green frames. The cysteine residues are shown in red. (**b**) Agarose gel electrophoresis diagram of *hdh-myostatin*.

**Figure 2:**
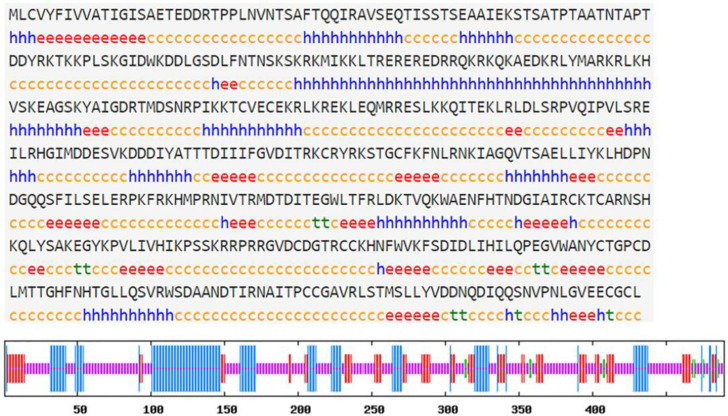
Amino acid secondary structure of hdh-myostatin. Hh expresses the α-helix. Ee expresses the extended strand. Tt expresses the β-turn. Cc expresses the random coil.

**Figure 3:**
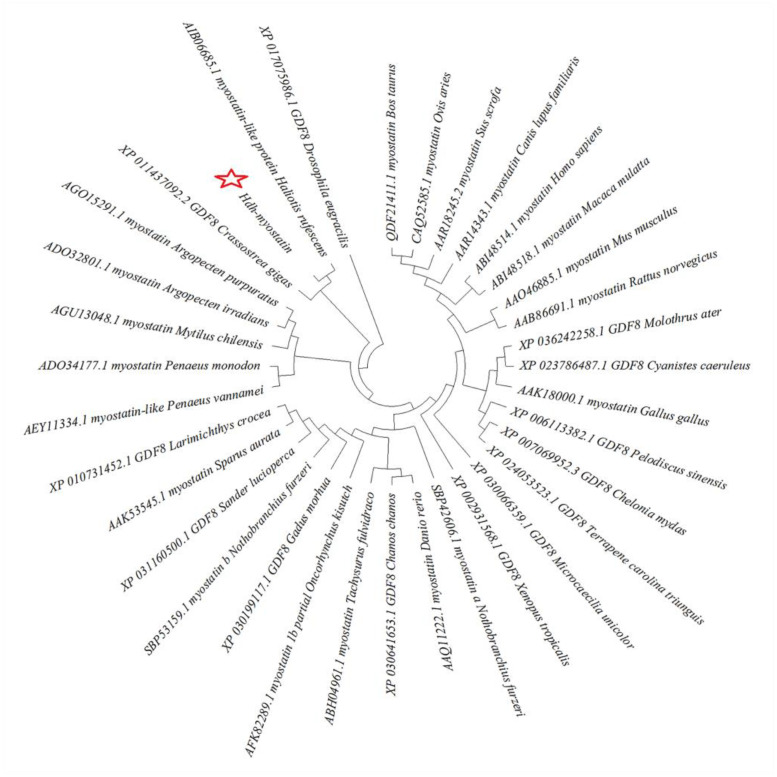
Phylogenetic tree based on amino acid homology of myostatin from vertebrates and invertebrates. A phylogenetic tree analysis was performed using the neighbor-joining algorithm using the MEGA program. Hdh-myostatin in this study is highlighted with a pentagram.

The full-length ORF of hdh-myostatin was obtained from the cerebral ganglion of *H. discus hannai*. The ORF sequence of hdh-myostatin comprises 1470 bp, encoding 489 amino acids. The protein sequence had an estimated molecular weight of 56.043 kDa and a theoretical pl of 9.309. The functional domains for hdh-myostatin included three parts, a putative signal peptide of 16 amino acids (MLCVYFIVVATIGISA) at the N-terminal region, a TGF-β propeptide of 186 amino acids (168–353 aa), and a mature TGF-β peptide of 104 amino acids (385–488 aa) at the C-terminal region. Three proteolytic processing sites, RQKR, RYRK, and RPRR, are marked with green frames in Figure 1. The C-terminal region of hdh-myostatin also contained nine highly conserved cysteine residues that are shown in red (Figure 1). The hdh-myostatin sequence was predicted to possess 25.97% α-helix, 16.56% extended strand, 2.04% β-turns, and 55.42% random coils in the secondary structure (Figure 2). The phylogenetic analysis of myostatin revealed the close relationship among abalones, with the present species clustering together with *Crassostrea gigas* and forming an independent branch (Figure 3). All molluscan myostatin sequences were grouped into one independent clade. This suggests evolutionary conservation in the sequence and structure of myostatin proteins in shellfish.

### 3.2. Expression Analysis of Hdh-Myostatin

**Figure 4:**
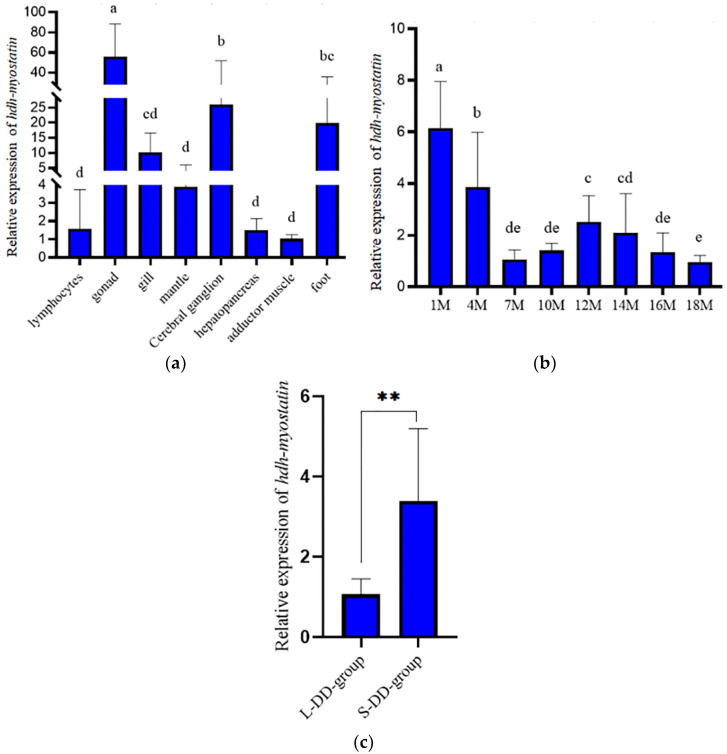
Expression pattern of *hdh-myostatin* from *H*. *discus hannai*. (**a**) Expression pattern of *hdh-myostatin* during various tissues. (**b**) Expression pattern of *hdh-myostatin* during development. (**c**) Expression pattern of *hdh-myostatin* in the fast-growing (L-DD-group) and slow-growing (S-DD-group) groups. Bars with different letters indicate significant differences (*p* < 0.05). ** indicate significant differences (*p* < 0.01).

As shown in Figure 4a, qRT-PCR showed that the *hdh-myostatin* mRNA was widely expressed at different levels in all of the examined tissues of *H. discus hannai*. The *hdh-myostatin* mRNA was expressed significantly higher in the gonad than in the other tissues of *H. discus hannai* (*p* < 0.05). The lowest levels of *hdh-myostatin* mRNA were observed in the lymphocytes, mantle, hepatopancreas, and adductor muscle. The expression level of *hdh-myostatin* mRNA presented a wide distribution of expression at different stages and lower expression levels in the later stages of development (Figure 4b). The expression level of *hdh-myostatin* mRNA was higher in the S-DD-group than in the L-DD-group (Figure 4c).

### 3.3. Growth-Related SNP Loci in Hdh-Myostatin

Fifteen SNPs were detected in total. The average PIC was 0.248, indicating a low level of polymorphism (PIC < 0.25). The result of association analysis indicated that nine SNPs (Table 2) from the CDS region of *hdh-myostatin* were significantly associated with growth traits in *H*. *discus hannai* (*p* < 0.05). As shown in Table 2, the growth traits for the CC and GC genotypes were superior to those of the GG genotype at the C-6G locus; the increased traits were shell length, shell width, total weight, and muscle weight (*p* < 0.05). The growth traits for the AA and GA genotypes were superior to those of the GG genotype at the A-117G locus (*p* < 0.05). The shell length of GG was longer than that of abalones with the AG genotype at the G-288A locus (*p* < 0.05). The growth traits for the CC genotype were superior to those for the AA genotype at the C-414A locus (*p* < 0.05). Moreover, all of the growth traits were significantly different (shell length, total weight, and muscle weight; *p* < 0.05), with only shell width not showing a significant difference for the T-437C locus. The shell width and total weight of abalones with the genotype GG were significantly larger than those with the genotype AG at the G-897A locus (*p* < 0.05).

### 3.4. Effects of Hdh-Myostatin dsRNA Injection

**Figure 5:**
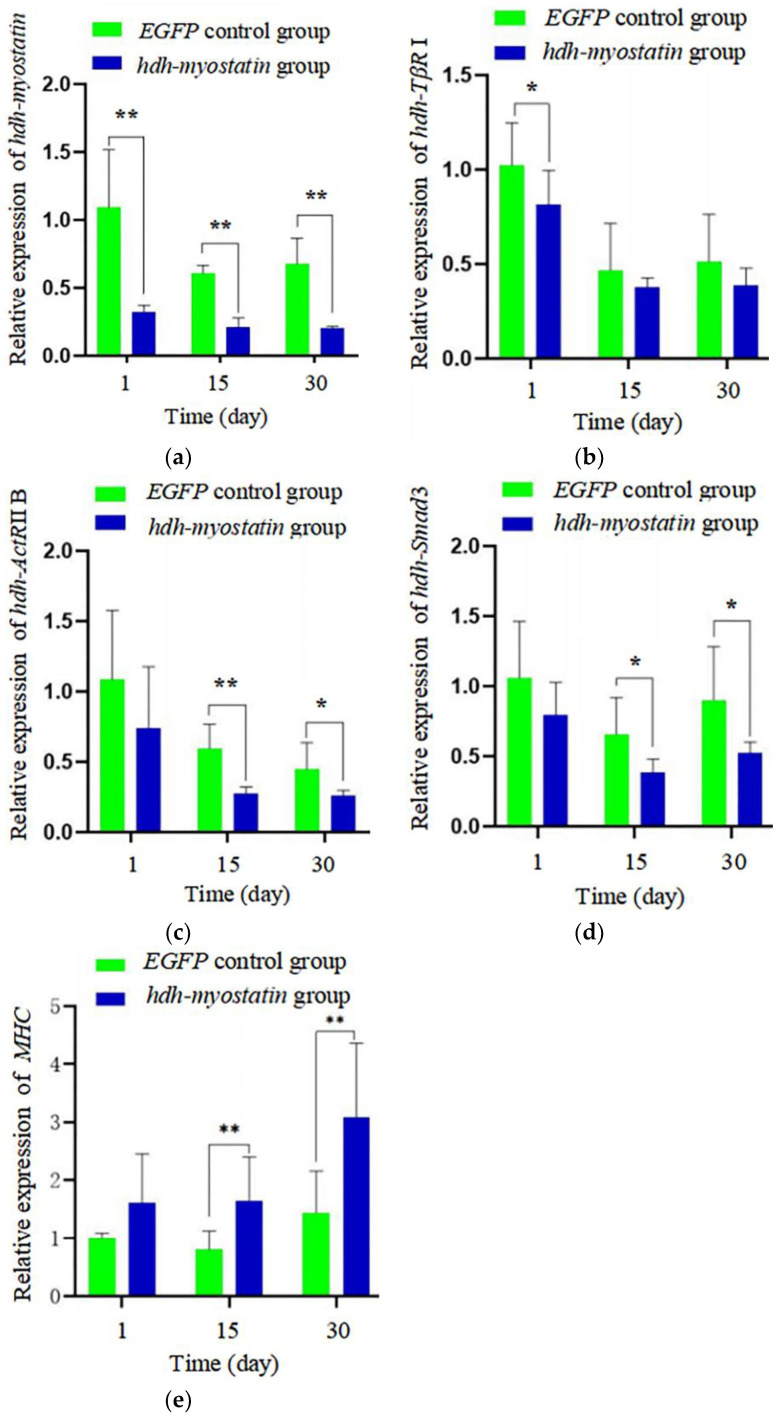
The results of qRT-PCR. (**a**) Relative expression level of *hdh-myostatin* after RNAi (RNA interference) in the cerebral ganglion. (**b**) Relative expression level of *hdh-TβR* I after RNAi in adductor muscle. (**c**) Relative expression level of *hdh-ActR* IIB after RNAi in adductor muscles. (**d**) Relative expression level of *hdh-Smad*3 after RNAi in adductor muscle. (**e**) Relative expression level of *MHC* after RNAi in adductor muscles. The expression of target genes was normalized to the *18S rRNA* and *β-actin* gene as the internal reference. These results are shown as mean values ± SD. Significant differences in gene expression levels are shown as ** *p* < 0.01 and * *p* < 0.05.

Compared with the *EGFP* control group, the expression levels of *hdh-myostatin* in the cerebral ganglion decreased by 60–70% (Figure 5a) after being treated with *hdh-myostatin* dsRNA at days 1, 15, and 30 (*p* < 0.05). After being treated with *hdh-myostatin* dsRNA at day 1, the mRNA expression of *hdh-TβR* I in the adductor muscle significantly decreased compared with the control group (Figure 5b) (*p* < 0.05), but the expression levels of *hdh-ActR* IIB (Figure 5c), *hdh-Smad*3 (Figure 5d), and *MHC* (Figure 5e) were not significantly different (*p* > 0.05). After being treated with *hdh-myostatin* dsRNA at days 15 and 30, the mRNA expression of *hdh-TβR* I in adductor muscle was not significantly different (Figure 5b) (*p* > 0.05), but the expression levels of *hdh-ActR* IIB (Figure 5c) and *hdh-Smad*3 (Figure 5d) significantly decreased (*p* < 0.05 and *p* < 0.01), and the expression levels of *MHC* significantly increased (Figure 5e) (*p* < 0.01).

After one month of the RNAi experiment, the increments of shell length and total weight in the experimental group (treated with *hdh-myostatin* dsRNA) were significantly higher than in the control group (treated with *EGFP* dsRNA, *p* < 0.05). However, the increment of shell width in the experimental group was slightly higher than that of the *EGFP* control group (*p* > 0.05) (Table 3).

### 3.5. Verification in the Hybrid Lvpan Abalone

**Figure 6:**
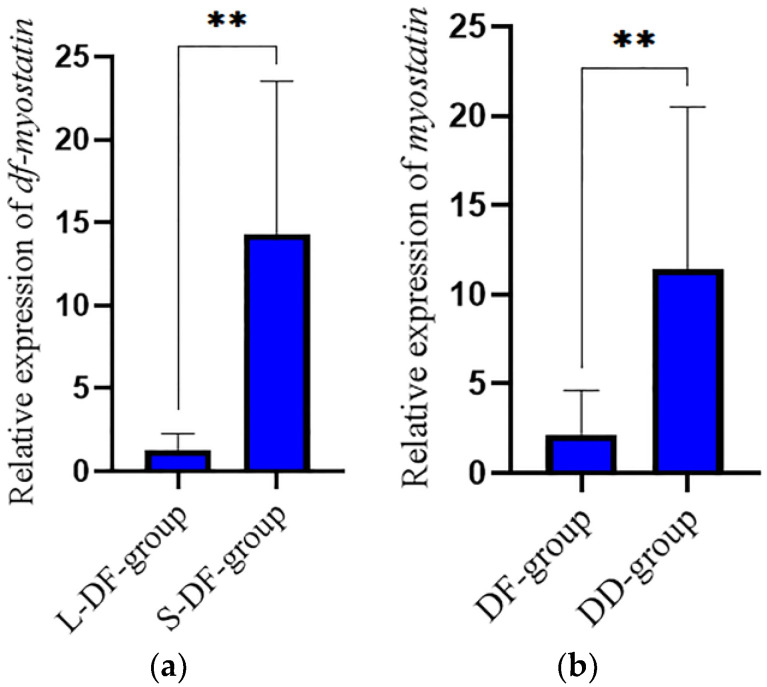
Expression pattern of *myostatin* mRNA. (**a**) Expression of *hdh-myostatin* in adductor muscle between fast-growing (L-DF-group) and slow-growing (S-DF-group) groups. (**b**) Expression of *myostatin* in adductor muscle between *H. discus hannai* (DD) and lvpan abalone (DF). ** indicate significant differences (*p* < 0.01).

As shown in Figure 6, the expression level of Lvpan abalone *df-myostatin* mRNA was higher in the S-DF-group than in the L-DF-group (*p* < 0.05). In addition, the expression level of *myostatin* mRNA was higher in the *H. discus hannai* group (DD-group) than in the Lvpan abalone group (DF-group) (*p* < 0.05).

## 4. Discussion

Myostatin is one of the most important members of the TGF-β superfamily. In this study, the CDS region of hdh-myostatin was cloned from the adductor muscle of *H. discus hannai* and comprised 1470 bp encoding 489 amino acids. The amino acid sequence contained an N-terminal signal peptide and a C-terminal mature TGF-β peptide. These results were fully consistent with the typical protein structural characteristics of the TGF-β superfamily, as demonstrated in studies with *Chlamys nobilis* [20] and *H. rufescens* [21]. The homology results showed that functional elements of the myostatin amino acid sequences were highly conserved among different species, suggesting that the function of myostatin was conserved. The result of the phylogenetic analysis showed that hdh-myostatin and myostatin of *H. rufescens* were clustered, forming a separate branch, and then clustered with myostatin of *Crassostrea gigas*, indicating that the relationship between the two was relatively close, and the sequence was furthest from those of mammals such as cattle, sheep, and pigs.

In terms of the expression in different tissues, *myostatin* in mammals is primarily expressed in skeletal muscle, with minimal or no expression in other tissues [29]. In fish, *myostatin* is more widely distributed [30]. For example, it is expressed in different tissues of zebrafish [31], with the highest expression in muscle. The *myostatin* of rainbow trout is not only highly expressed in muscle but also in brain, testis, eyes, and spleen [32]. The tissue expression of *myostatin* in shellfish is similar to that in fish, and it is expressed in different tissues [19]. The *myostatin* is expressed in the intestine, muscle, mantle, and cephalic ganglion of *H. rufescens* [21]. In this study, *hdh-myostatin* was widely distributed in all tissues, but the relative expression level was the highest in the gonad, followed by the cerebral ganglion and foot, while there was no significant difference in the expression levels in the adductor muscle, gill, mantle, or other tissues. This was not fully consistent with the results from other shellfish. However, *hdh-myostatin* expression of *H. discus hannai* was similar to that of amphioxus [33], where the protein was highly expressed in other tissues besides muscle. We suspect that the effect of *hdh-myostatin* regulation of growth may involve mechanisms other than an autocrine manner, possibly through the neuroendocrine system, similar to the mechanism whereby *myostatin* inhibits muscle growth by regulating pituitary development or IGF1 in vertebrates [34,35]. These results suggest that the function of *hdh-myostatin* in *H. discus hannai* may not be limited to muscle pattern formation but instead may also be involved in other biological processes such as gonad development. In terms of expression during different developmental stages, *hdh-myostatin* was expressed at all stages of development in *H. discus hannai*, with significant differences at different months of age, similar to the results of Wang et al., (2005) [36]. The relative expression of *hdh-myostatin* was highest at one month of age, and its expression showed a trend of fluctuation and downregulation with the increase in development, suggesting that *hdh-myostatin* may regulate the muscle growth of *H. discus hannai*.

The SNPs related to growth traits in the myostatin gene can be used in marker-assisted selective breeding. For example, TC genotypes of the dry body weight in the *Apostichopus japonicus* myostatin gene were significantly higher than those with CC genotypes [37]. To further study the function of *hdh-myostatin* in *H. discus hannai*, we analyzed the polymorphism of *hdh-myostatin* and the correlations with growth traits. The results showed that there were 15 SNPs in the region of the ORF of *hdh-myostatin* in *H. discus hannai*, among which nine SNPs were significantly correlated with growth traits. The results suggested that *hdh-myostatin* is closely related to the growth traits of *H. discus hannai* and that it participates in the growth regulation process. The myostatin gene has also been confirmed to be closely related to growth in economically important shellfish such as *Sinonovacula constricta* [19], *Chlamys farreri* [38], and *H. diversicolor supertexta* [10]. These SNPs can be used as candidate molecular markers for growth-related marker-assisted selective breeding of *H. discus hannai*.

The myostatin gene inhibits the growth and development of animal muscle by controlling the number, size, and proliferation of muscle cells [39]. Inhibition of *myostatin* expression by RNAi can increase muscle content [40], as shown in mammals, fish [41], crustaceans [42], and the Yesso scallop [14]. Chen et al., (2006) found that the growth traits in the DNA-injected group were significantly greater than those of the control group by directly injecting exogenous gene fragments into the genome of *H. diversicolor supertexta* [43]. In this study, we successfully inhibited the expression of *hdh-myostatin* by directly injecting dsRNA into the *H. discus hannai*, resulting in significant increases in shell length, growth, and total weight gain compared with the control group, further demonstrating that *hdh-myostatin* can inhibit the growth of mollusks.

In addition, it has been reported that *hdh-myostatin* mediates signal transmission through type I and type II receptors. The activated *hdh-myostatin* first binds to the receptor *ActR* IIB and then phosphorylates type I receptor *TβR* I (*ALK*4/5) to form a complex, thereby acting to inhibit cell proliferation and differentiation [23,44]. When *hdh-myostatin* expression was inhibited, the expression levels of *hdh-TβR* I and *hdh-ActR* IIB responded to some extent. After 15 and 30 days of *hdh-myostatin* interference, the expression levels of *hdh-ActR* IIB were significantly lower than in the control group. The expression level of *hdh-TβR* I was not significantly different from that of the control group, suggesting that *hdh-ActR* IIB is the *hdh-myostatin* receptor of *H. discus hannai* and that other *hdh-myostatin* type I receptors may also bind to it.

Myostatin binds with high affinity to the receptor ActR IIB, which in turn initiates signaling through a smad2/3-dependent pathway [45,46]. When myostatin, ActR IIB, and TβR I form a complex, phosphorylated Smad2/3 and Smad4 enter the nucleus to activate the transcription of target genes and inhibit muscle growth [47,48]. In this study, after 15 and 30 days of *hdh-myostatin* interference, the expression level of *hdh-Smad*3 in adductor muscle was significantly lower than in the control group, while the expression level of *MHC* significantly increased, suggesting that *hdh-myostatin* inhibited the muscle growth of *H. discus hannai* and that this effect was achieved through signal transmission by smad3.

Finally, we verified the function of *myostatin* in the hybrid Lvpan abalone and found that the expression in the larger abalones in DD (*H. discus hannai*) and DF (Lvpan abalone) at the same age was lower than that in smaller abalones. The *myostatin* expression of DF was lower than that of DD at the same age, further indicating that *myostatin* played a negative role in regulating the growth of abalone. In *Fenneropenaeus merguiensis*, a higher expression level of *FmMstn* was also observed in smaller shrimp of the same age [42].

## 5. Conclusions

In this paper, we cloned and characterized the CDS sequence of *hdh-myostatin* from the cerebral ganglion of *H. discus hannai*. The hdh-myostatin possesses the N-terminal regions of the TGF-β propeptide and mature TGF-β peptide, and it belongs to the TGF-β superfamily. The results of qRT-PCR indicated that *hdh-myostatin* mRNA was widely expressed in different tissues of *H*. *discus hannai*. There were nine SNPs from the CDS region of *hdh-myostatin* that were significantly associated with growth traits. The result of *hdh-myostatin* interference demonstrated that it could affect the growth of *H*. *discus hannai*. Our findings have provided a foundation for further exploring the functions of TGF-β superfamily ligand members in the growth process of abalone, as well as a reference for the application of molecular markers for growth traits in the breeding of shellfish.

## Figures and Tables

**Table 1:** Sequences of the primer pairs used in this study.

Primer	Sequence (5′-3′)
*hdh-myostatin*-F	AGTGTATTGGCAAGTCGTGA
*hdh-myostatin*-R	CAACGGCAGTATAGTAGGTCAA
*hdh-myostatin*-dsF	TAATACGACTCACTATAGGGGCCGGTCCTCATCGTTCAC
*hdh-myostatin*-dsR	TAATACGACTCACTATAGGGTTACAAGCACCCACATTCTTCCAC
*EGFP*-dsF	TAATACGACTCACTATAGGGGTGCCCATCCTGGTCGAGCT
*EGFP*-dsR	TAATACGACTCACTATAGGGTGCACGCTGCCGTCCTCGAT
*hdh-myostatin*-qF	TGAGTCGGGAGATTCTTCGC
*hdh-myostatin*-qR	TGATGATGTCGGTTGTCGTG
*hdh-TβR* I-qF	ACCATCACACCATGACACAG
*hdh-TβR* I-qR	GCCACACCTCACCGTACCTC
*hdh-ActR* IIB-qF	GCTGGTAATGAAGGGCTG
*hdh-ActR* IIB-qR	AGTCGTGATGGGAAGTTG
*hdh-Smad*3-qF	GTTTGCCGAGTGTCTCAGTG
*hdh-Smad*3-qR	CCCTGGTGGTATCTTGCAGA
*MHC*-qF	GACCCCAACGACCCTGATAT
*MHC*-qR	TCTTCTCCCTTGGTGCTCTG
*β-actin*-qF	GGTATCCTCACCCTCAAGT
*β-actin*-qR	GGGTCATCTTTTCACGGTTG
*18S rRNA*-qF	TTCCCAGTAAGCGTCAGTCATC
*18S rRNA*-qR	CGAGGGTCTCACTAAACCATTC

**Table 2:** Correlation of SNPs in *hdh-myostatin* with growth traits in the *Haliotis discus hannai* (mean ± SD).

Locus	Genotype	Sample Number	Shell Length (mm)	Shell Width (mm)	Total Weight (g)	Muscle Weight (g)
C-6G	CC	102	74.37 ± 9.68 ^a^	49.85 ± 6.40 ^a^	42.54 ± 16.13 ^a^	17.58 ± 7.58 ^a^
	GC	83	75.52 ± 9.53 ^a^	50.66 ± 5.90 ^a^	43.82 ± 16.07 ^a^	17.96 ± 7.86 ^a^
	GG	32	68.34 ± 7.84 ^b^	47.15 ± 5.31 ^b^	33.48 ± 10.20 ^b^	13.59 ± 5.05 ^b^
T-115C	TT	102	74.38 ± 9.66 ^a^	49.93 ± 6.38 ^ab^	42.52 ± 16.06 ^a^	17.44 ± 7.60 ^a^
	CT	82	75.16 ± 9.69 ^ab^	50.45 ± 6.11 ^a^	43.56 ± 16.42 ^a^	18.03 ± 7.97 ^a^
	CC	34	69.72 ± 8.42 ^c^	47.78 ± 5.39 ^b^	35.03 ± 11.39 ^b^	14.09 ± 5.39 ^b^
A-117G	AA	102	74.55 ± 9.65 ^a^	50.02 ± 6.36 ^ab^	42.89 ± 16.00 ^a^	17.65 ± 7.60 ^a^
	GA	81	75.07 ± 9.71 ^a^	50.41 ± 6.13 ^a^	43.36 ± 16.42 ^a^	17.91 ± 7.94 ^a^
	GG	34	69.72 ± 8.42 ^b^	47.78 ± 5.39 ^b^	35.03 ± 11.39 ^b^	14.09 ± 5.39 ^b^
C-282T	CC	128	74.18 ± 9.85 ^a^	49.97 ± 6.52 ^a^	42.55 ± 16.47 ^a^	17.45 ± 7.69 ^a^
	TC	71	74.16 ± 9.51 ^a^	49.88 ± 5.65 ^a^	41.98 ± 15.28 ^ab^	17.28 ± 7.62 ^a^
	TT	20	69.95 ± 8.00 ^a^	47.12 ± 5.29 ^a^	34.39 ± 11.65 ^b^	14.10 ± 5.78 ^a^
G-288A	GG	187	74.56 ± 9.15 ^a^	49.92 ± 5.97 ^a^	42.40 ± 15.41 ^a^	17.43 ± 7.41 ^a^
	AG	29	69.49 ± 11.35 ^b^	48.48 ± 7.29 ^a^	37.37 ± 18.03 ^a^	15.44 ± 8.29 ^a^
C-414A	CC	101	74.95 ± 9.63 ^a^	50.36 ± 6.36 ^a^	43.53 ± 16.01 ^a^	17.84 ± 7.56 ^a^
	AC	82	74.03 ± 9.56 ^ab^	49.59 ± 5.93 ^ab^	41.38 ± 15.84 ^ab^	17.01 ± 7.72 ^ab^
	AA	35	69.67 ± 8.97 ^b^	47.64 ± 5.79 ^b^	35.26 ± 13.00 ^b^	14.51 ± 6.32 ^b^
T-437C	TT	118	74.01 ± 10.14 ^a^	49.83 ± 6.58 ^a^	42.25 ± 16.67 ^a^	17.37 ± 7.78 ^a^
	CT	80	74.41 ± 9.45 ^a^	49.90 ± 5.82 ^a^	41.86 ± 15.01 ^a^	17.16 ± 7.44 ^ab^
	CC	19	69.13 ± 5.67 ^b^	47.10 ± 4.37 ^a^	33.28 ± 9.26 ^b^	13.47 ± 4.57 ^b^
G-897A	GG	179	73.09 ± 9.68 ^a^	49.28 ± 6.16 ^a^	40.53 ± 15.75 ^a^	16.71 ± 7.61 ^a^
	AG	37	76.87 ± 8.81 ^b^	51.41 ± 5.88 ^a^	46.45 ± 15.92 ^b^	18.79 ± 7.31 ^a^
G-1278A	GG	143	74.50 ± 9.57 ^a^	50.09 ± 5.98 ^a^	42.15 ± 15.16 ^a^	17.20 ± 7.15 ^a^
	AG	69	73.52 ± 9.50 ^ab^	49.52 ± 6.51 ^a^	41.70 ± 16.99 ^a^	17.43 ± 8.30 ^a^
	AA	8	65.00 ± 8.68 ^b^	44.86 ± 5.58 ^b^	31.15 ± 14.02 ^a^	12.28 ± 6.34 ^a^

Note: Mean values with different letters within a column are significantly different (*p* < 0.05).

**Table 3:** The growth of *Haliotis discus hannai* after *hdh-myostatin* RNA interference (mean ± SD).

Indicator	*EGFP* Control Group (*N* = 39)	*hdh-Myostatin* Experimental Group (*N* = 38)
Initial shell length (mm)	35.48 ± 1.08 ^a^	35.19 ± 1.10 ^a^
Initial shell width (mm)	23.45 ± 0.92 ^a^	23.51 ± 0.78 ^a^
Initial total weight (g)	5.76 ± 0.48 ^a^	5.69 ± 0.51 ^a^
Final shell length (mm)	37.32 ± 1.17 ^a^	37.45 ± 1.48 ^a^
Final shell width (mm)	24.83 ± 0.81 ^a^	25.25 ± 0.92 ^a^
Final total weight (g)	6.57 ± 0.59 ^a^	6.69 ± 0.52 ^a^
increment of shell length (mm)	1.87 ± 0.46 ^a^	2.23 ± 0.66 ^b^
increment of shell width (mm)	1.51 ± 0.39 ^a^	1.75 ± 0.48 ^a^
increment of total weight (g)	0.81 ± 0.31 ^a^	1.08 ± 0.40 ^b^

Note: Mean values with different letters within a column are significantly different (*p* < 0.05).

## Data Availability

The data presented in this study are available upon request from the corresponding authors.
